# Team Training (Training at Own Facility) versus Individual Surgeon's Training (Training at Trainer's Facility) When Implementing a New Surgical Technique: Example from the ONSTEP Inguinal Hernia Repair

**DOI:** 10.1155/2014/762761

**Published:** 2014-11-19

**Authors:** Jacob Rosenberg, Kristoffer Andresen, Jannie Laursen

**Affiliations:** Department of Surgery, Herlev Hospital, University of Copenhagen, Herlev Ringvej 75, 2730 Herlev, Denmark

## Abstract

*Background.* When implementing a new surgical technique, the best method for didactic learning has not been settled. There are basically two scenarios: the trainee goes to the teacher's clinic and learns the new technique hands-on, or the teacher goes to the trainee's clinic and performs the teaching there. *Methods.* An informal literature review was conducted to provide a basis for discussing pros and cons. We also wanted to discuss how many surgeons can be trained in a day and the importance of the demand for a new surgical procedure to ensure a high adoption rate and finally to apply these issues on a discussion of barriers for adoption of the new ONSTEP technique for inguinal hernia repair after initial training. *Results and Conclusions.* The optimal training method would include moving the teacher to the trainee's department to obtain team-training effects simultaneous with surgical technical training of the trainee surgeon. The training should also include a theoretical presentation and discussion along with the practical training. Importantly, the training visit should probably be followed by a scheduled visit to clear misunderstandings and fine-tune the technique after an initial self-learning period.

## 1. Introduction

Over the years, numerous new surgical techniques have been implemented [1], but there is no overall consensus of how to facilitate this process. Typically, the trainee surgeon will visit another department and watch a procedure or he/she will see it in videos either at international meetings or on the Internet. Thereafter, a sort of trial and error phase will follow in the surgeon's own department until the technique is running smoothly. This scenario may not be optimal, since a formalized training program would probably result in better learning and thereby better patient outcome.

Traditionally, learning has been defined as a process with creation of new skills and knowledge at an individual level. Over the recent years, this approach has been questioned. Research has shown that high-standard learning should be seen as a social process, where the interaction between individuals creates new learning [2]. Human learning should therefore be seen as a social interaction between the individual and his environment [2].

The success rate when implementing a new surgical technique may be measured by the percentage of the trainee surgeons who will perform the new procedure when they are not in the training situation anymore. Thus, when spending resources on instruction and training, a high adoption rate would be optimal. The training setup could affect this adoption rate and different setups for instruction trainings can be discussed. The trainee might visit the trainer and take the new technique to his own department by himself. Another model implies two trainees visiting the trainer simultaneously. Two trainees from the same department can then help each other establishing the new technique when they get home. It has been shown that when unsupervised training is performed in groups of two (dyad training), the performance is rated better compared with individual training and the trainees rate their level of confidence higher [3, 4]. The trainer could also visit a department where he/she will not only train one or two surgeons, but also the entire OR-team since a good team performance (i.e., surgeons and other OR-personnel) has been shown to enhance patient safety and a poor team performance has been linked to higher risk of complications [5]. Furthermore, a setup with a course followed by expert proctoring was found to be the preferred method of learning when studying barriers in the adoption of another type of hernia surgery [6].

The current adoption rate after training in the ONSTEP inguinal hernia repair technique [7] is 57% across Europe (see Table 1) and we therefore want to discuss different training scenarios in order to preferably increase the adoption rate for this new technique. The different training methods can be applied also for other new surgical techniques and are therefore of broader relevance. Thus, the aim of this paper was to discuss different training scenarios when implementing the ONSTEP technique for inguinal hernia repair with a focus on either moving the trainee to the teacher's department or moving the teacher to the trainee's department.

## 2. Moving the Trainee

A formal educational program has been developed for training surgeons in the ONSTEP technique for inguinal hernia repair. This implies that the trainees are visiting the teacher's department where typically five primary inguinal hernia repairs are scheduled for the training session. Typically, four visiting surgeons will participate and on the day of surgery the teacher will perform the first procedure with the trainees as assistants and thereafter the trainees will perform one case each with the teacher as an assistant. Before going to the OR, typically the night before, there will be a theoretical teaching session, where the trainees are presented with a slide show, technical videos, and detailed discussion of pros and cons of the new procedure. The trainees get written material covering the background and detailed descriptions with drawings of the surgical procedure as well as a surgical technique instructional video. After the day of surgery, the trainees go back to their own departments and there are no formal follow-up visits planned in the training program.

## 3. Moving the Teacher to the Trainee's Department

Another method is to use proctoring where the teacher is visiting the trainee's department. Typically, the teacher will arrive the day before surgery and the same theoretical session would be performed on the evening before surgical operations. Here one or two trainees, typically together with OR-nurses and anesthetists, will be shown slides explaining the rationale for the new operative procedure as well as a thorough discussion of the technical steps of the operation. The next day, operations will be performed in the trainee's department and the same people will be present in the operating room as in the evening before. Again, the teacher will perform the first procedure with the trainee as assistant and trainees will do the next procedures with the teacher as an assistant. After the day of surgery, the teacher will go back to his own department and formal follow-up is again not scheduled routinely.

## 4. Pros and Cons of Visiting Learning Facility

Most perspectives of organizational learning have taken one of two approaches either focusing on the individual learning or on individual learning as a model for organizational action. However, learning cannot be understood in such a narrow understanding. A central issue in the debates about the relation between the social world and the individual social acting has been about how individuals reinforce and redefine their understanding of knowledge [8]. A study in Finland showed that the ability to learn was linked to practical experiences, where a good atmosphere and a sense of being in a community of practice were important components [9]. Learning surgeons new techniques would concern the relations between the surgeon's thinking, his action, and at the same time the social sphere, where the new techniques should be developed [8, 10]. Recent studies have suggested that to overcome some of these challenges in gaining new techniques and skills, it would be advantageous to use supervisor support in own workplace [8, 10].

**Figure 1:**
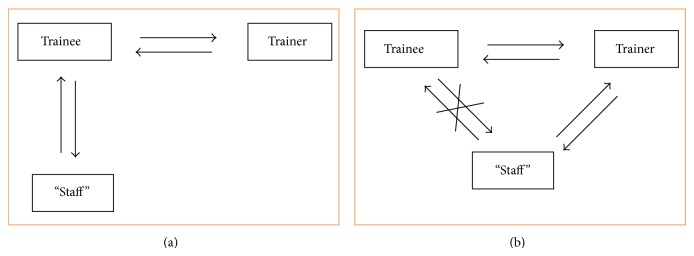
In scenario (a), the trainee visits the trainer. The trainee has to answer all the questions and work with the skepticism from his department/staff when he gets back to his own workplace. In scenario (b), the situation is different because the trainer is present at the trainees department where he/she can answer questions, thus relieving the trainee and allowing him/her to focus on the technique.

Team training can have beneficial effects on several factors in the OR such as attitude and communication [11]. The attitude and communication in the OR could influence the adoption rate, since one of the problems that arises when you have the trainee visiting a training center is that the trainee will have a lot of questions that need to be answered and this can be done in a direct dialog with the teacher, but when the trainee goes back to his own department, the trainee will have to answer all the questions that arise from the staff (Figure 1). These questions can be related to what kind of instruments that would be appropriate for the procedure, what kind of anesthesia is most appropriate, indications for the surgical procedure, and so forth. This will put stress on the trainee when he is alone because he has to focus on both remembering and performing the ONSTEP procedure correctly and also at the same time he will have to answer all the questions that arise from the staff.

As seen in Figure 1, the situation is different when the trainer and the trainee are at the trainee's department. In this situation, the trainee can focus on performing and learning the ONSTEP technique while at the same time the staff that also has their first exposure to the technique can ask all the questions to the trainer. The trainer can, by answering questions, help to relieve the skepticism that might arise in the department when a new technique is introduced. This could possibly give a higher adoption rate afterwards, since the staff will then already know what to do, critical questions have been answered, and the trainee can focus on performing the technique. Furthermore, when the staff experiences that the technique is feasible and relevant for their patients, they will expect the new procedure to be implemented thus putting pressure on the trainee to continue with the new surgical procedure. Furthermore, the involvement and training of the OR team as a whole have been found to enhance the implementation of new surgical techniques [12].

When the trainer visits the trainee, the trainer will have to adapt to the way the trainee's department is organized. The trainer might not know exactly which patients have been booked for demonstration and training, and the trainer will have to also teach the OR and perioperative staff about the new technique. This might be a difficult and stressful task for the trainer, but the expected effect of the training may be higher.

## 5. How Many Surgeons Can Be Trained in a Training Day?

Obviously, the number of trainees in a given training session will have an impact on the learning effect, especially when training practical surgical techniques. Thus, it will be beneficial to have as few as possible for a training session. However, there is a clear advantage of training at least 2 surgeons from the same department because they can use each other for discussions and support during the subsequent implementation period.

## 6. Follow-Up

In the current training scenarios as used in the implementation of the ONSTEP technique for inguinal hernia repair, there have been no formal follow-up visits after the training session. This may not be optimal. Follow-up visits might enhance the motivation to perform the technique afterwards and also give an initial higher learning outcome, since the follow-up might be seen as test [13].

**Figure 2:**
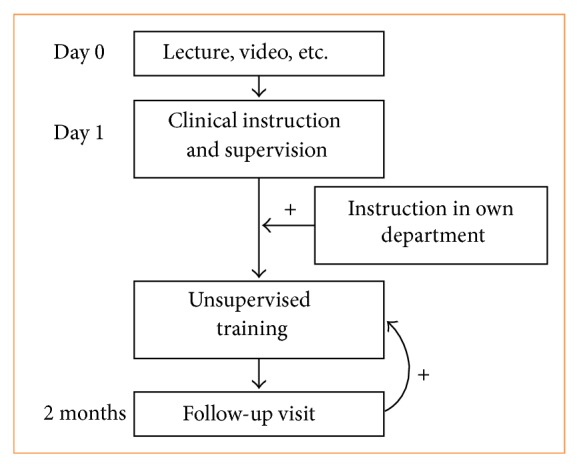
The proposed training program with follow-up. The transition from supervised training to unsupervised training might be facilitated when instruction is done at the trainees department. A scheduled follow-up visit might also have a positive feedback on the initiation and continuing of the unsupervised training.

There seems to be a need for some kind of repetition or follow-up visits with the opportunity to either discuss the problems or better to repeat the hands-on teaching session in order to clear smaller misunderstandings and to fine-tune the surgical technique. Furthermore, if a follow-up visit is formally scheduled after, for example, 20 procedures and after maybe 2 months, the adoption rate will be secured because the trainee will be expected to do the required 20 procedures before the follow-up session (Figure 2), and thereby scheduled follow-up visits have a positive feedback on the unsupervised training. Ideally these follow-up visits should facilitate the trainees reflection on his or her performance and learning, in order to increase the learning outcome [14]. Scheduled follow-up can be implemented both where the trainee visits the teacher and where the teacher visits the trainee.

## 7. Importance of the Demand for a New Surgical Procedure

It is obvious that it is almost impossible to implement a new surgical procedure if a demand from a clinical point of view is not clear. In the case of the ONSTEP technique for inguinal repair it is, however, an easy discussion because there are problems with chronic pain after the most commonly used technique that in most countries is the Lichtenstein technique [15–17]. Thus, around 16% of patients after the Lichtenstein procedure will experience chronic pain that inhibits normal daily activities [18]. In the Lichtenstein technique, the hernia repair is performed by an anterior approach where a synthetic mesh is implemented in the inguinal region anterior to the muscle layers [19]. In this area, the major nerves are present and this may be a contributing factor to the pathophysiology of the development of chronic pain after Lichtenstein procedure [20]. In the ONSTEP procedure, the dissection is performed in a different plane, where the major nerves are not hit either by the dissection or by the mesh itself [7]. Furthermore, the mesh is not fixated with sutures or staples to the tissue, which also may be a contributing factor for the observed extremely low rate (0%) of chronic pain after the ONSTEP procedure [7]. Thus, the clinical arguments for implementing ONSTEP instead of Lichtenstein seem obvious for surgeons with knowledge within this clinical field. In the training plan, it is with every new surgical technique important to first of all explain the rationale for using another technique rather than the usual one. Therefore, this information is included in the training material at the first day of training with a theoretical overview given by the teacher to the trainees.

## 8. Barriers for Adoption of the ONSTEP Technique after Initial Training

The barrier for increasing the adoption rate to a high level may not be a single factor. It is our impression, although not supported by concrete data, that there is a barrier when the trainee gets back to his own department and should continue the self-training. In this scenario, the trainee is the only one who has the motivation for starting this new procedure, and it takes some effort in an otherwise busy daily schedule to carry this new technique into the daily program. Furthermore, the trainee will not only have to focus on the surgical technique itself, but will also have to involve all the other partners in the perioperative team and explain to these people the pros and cons of this new procedure. Thus, the collective effort is high and only carried by the trainee.

Another barrier for a high adoption rate may be the lack of formal follow-up in the previously used training scenarios. Previously, follow-up was only performed by a sales representative from a mesh company and this may not fulfill all the needs of the trainee surgeon. It would therefore be beneficial to schedule a formal follow-up either by the trainee visiting the teacher or by the teacher visiting the trainee, and the best way would be to implement hands-on training again, where the technique can be fine-tuned and smaller problems and troubles can be solved. Furthermore, the knowledge of the trainee that the teacher will be present for a follow-up session will also increase the motivation for self-training in the period between the first and the second training sessions. Thereby, the adoption rate will probably be secured.

It may be a challenge for the trainee to implement the technique in his own department because of his responsibility of securing the correct instruments and teaching the surgical assistant about the procedure, the OR nurses, and the anesthesia team as well as the administrators of the department, since there may be some economic consequences also present when implementing a new surgical technique. Also, the perioperative team such as the nurses in recovery and the surgical ward needs education because the patient may present differently after operation compared with the usual cases.

## 9. Conclusion

The optimal situation, if practically possible, would probably be to move the teacher to the trainee's department in order to benefit from team-training effects when implementing the ONSTEP technique for inguinal hernia repair. By moving the teacher instead of the trainee, the entire perioperative team will receive the same information and the trainee surgeon may be able to focus more on the technical aspects of the surgical technique in the training phase rather than focusing on education of the entire team. Furthermore, it seems obvious that the educational program should include a theoretical discussion of pros and cons of the new technique, a rationale for implementing the new technique, and discussion of the technical steps and tips and tricks as well as hands-on training. This should be followed by a scheduled follow-up visit to clear minor technical misunderstandings and thereby fine-tune the surgical technique (Figure 2).

## Figures and Tables

**Table 1:** Activity for ONSTEP training across Europe 2013. A total of 46 workshops with 146 trainees from 13 different countries have been performed in 2013.

Country	Trainees
United Kingdom	6
Germany	22
France	8
Spain	14
Italy	13
Belgium	16
Denmark	16
Sweden	8
Finland	9
Greece	19
Austria	8
Switzerland	4
Czech Republic	3

Total Europe	146
